# Therapeutic effects of autologous hematopoietic stem cell transplantation in multiple myeloma patients

**DOI:** 10.3892/etm.2013.1261

**Published:** 2013-08-16

**Authors:** CHENGCHENG FU, JUAN WANG, XUE XIN, HUI LIU, SHENGLI XUE, XIAO MA, ZHENGMING JIN, AINING SUN, HUIYING QIU, DEPEI WU

**Affiliations:** Jiangsu Institute of Hematology, Key Laboratory of Thrombosis and Hemostasis, Ministry of Health, The First Affiliated Hospital of Soochow University, Suzhou, Jiangsu 215006, P.R. China

**Keywords:** multiple myeloma, autologous hematopoietic stem cell transplantation, prognosis

## Abstract

The present study aimed to evaluate the effect of autologous hematopoietic stem cell transplantation (ASCT) on the response and outcome of patients with multiple myeloma (MM) and to analyze the factors influencing the prognosis of the disease. Retrospective analysis was performed in 27 patients with MM who had been treated by ASCT (ASCT group) and 28 patients treated with combined chemotherapy only (non-ASCT group) from May 2004 to August 2011. The impact on the depth of response, progression-free survival (PFS) and overall survival (OS) times, as well as associated prognostic factors of patients with MM, were analyzed. All patients successfully underwent hematopoietic reconstruction without transplantation-related mortality. The complete remission (CR) rate of patients in the ASCT group significantly increased from 25.9% (7/27) before ASCT to 70.4% (19/27) following ASCT (P<0.01). The probability of OS for 5 years was 52.2% for the patients in the ASCT group and 33.1% for those in the non-ASCT group (P>0.05). Univariate analysis in the ASCT group demonstrated that maintenance and consolidation therapies were associated with significant increases in PFS (P=0.01) and OS (P<0.01) times. The present study demonstrated that ASCT further increases the CR rate, prolongs PFS time and potentially increases the OS time. Incorporation of these novel agents, including the protea-some inhibitor bortezomib and the immunomodulatory drugs thalidomide and lenalidomide, into the induction, consolidation and maintenance phases has optimized the anti-myeloma activity of ASCT.

## Introduction

Multiple myeloma (MM) is a type of malignant plasma cell proliferative disease (1). MM was first reported in 1848 and is characterized by the proliferation of malignant plasma cells and subsequent enrichment of monoclonal paraproteins. In patients with MM, antibody-forming cells, such as plasma cells, are malignant and may potentially result in unusual manifestations. The proliferation of plasma cells in patients with MM may affect the normal production of blood cells, resulting in leukopenia, anemia and various diseases, as the aberrant antibodies impair humoral immunity. The survival rate is higher in young patients with MM and lower in elderly patients.

MM may cause diseases ranging from those that are asymptomatic to those that are severely symptomatic with complications requiring treatment (2). Although treatments may benefit patients by resulting in a longer life, less pain and fewer complications, currently no effective cure for MM exists. However, advances in therapeutic treatments have helped to reduce the occurrence and severity of the adverse effects of MM.

The complete remission (CR) rate of traditional chemotherapy is <5% and the median survival time is 3 years (3). Autologous hematopoietic stem cell transplantation (ASCT) following high-dose chemotherapy significantly improves the CR rate and extends the progression-free survival (PFS) and overall survival (OS) times in patients with MM. Therefore, it is the first-line standard treatment program for patients <65 years old (4).

The clinical application of novel targeted agents, including thalidomide, bortezomib and lenalidomide, may alter the induction therapy and conditioning regimen prior to transplantation, maintenance and consolidation therapies following transplantation. Therefore, the present study reviewed the clinical data of 27 patients with MM who had been treated by ASCT. Through the comparison between patients with very good partial remission (VGPR) following concurrent introduction therapy and patients with VGPR following consolidation chemotherapy, the efficacy of ASCT treatment in patients with MM, was examined. In addition, the importance of combining ASCT and novel agents for the treatment of MM was explored.

## Materials and methods

### 

#### Patient information

Twenty-seven patients with MM who were treated by ASCT at The First Affiliated Hospital of Soochow University (Suzhou, China) from May 2004 to August 2011 were studied. Of the 27 patients, two presented with POEMS syndrome. Twenty-eight patients with MM, who had received induction chemotherapy at The First Affiliated Hospital of Soochow University and achieved at least a VGPR without ASCT treatment in the same period, served as the control group. All patients met the diagnostic criteria of the 2012 NCCN guidelines (5). The basic clinical patient information is shown in Table I and there were no significant differences between the two treatment groups in terms of gender, age, genotype and staging. The study was approved by the ethics committee of Soochow University (Suzhou, China). Prior written and informed consent was obtained from every patient.

### Treatment plan ASCT group

#### Induction chemotherapy

Eleven patients received traditional chemotherapy, which consisted of vincristine + doxorubicin/liposomal doxorubicin + dexamethasone (VAD/DVD); vincristine, melphalan, cyclophosphamide and prednisone (VMCP); and vincristine, BCNU, melphalan, cyclophosphamide and prednisone (M2). The other 16 patients received bortezomib + dexamethasone ± doxorubicin/liposomal doxorubicin (VD/PAD) as chemotherapy. The median number of chemotherapy treatments was four (range, 1–16). Following the induction therapy, there were eight cases of CR, 12 cases of non-CR (nCR), six cases of VGPR and one case of partial remission (PR).

#### Mobilization chemotherapy

Fourteen patients received mobilization chemotherapy and were treated with 3–4 g/m^2^ high-dose cyclophosphamide (HDCTX) and 5–10 g/kg/day granulocyte colony-stimulating factor (G-CSF). Moreover, 12 patients received G-CSF only and one patient switched to plerixafor (AMD3100) with G-CSF for one to three treatments following G-CSF mobilization failure. Of these, one patient demonstrated a concentration of CD34*^+^* cells of <1.0×10^6^/kg in the peripheral blood stem cell collection and the autologous bone marrow stem cells were collected. The collected median CD34*^+^* cell count was 2.89×10^6^/kg (range, 1.14−6.54×10^6^/kg).

#### Conditioning regimen

Eleven patients were treated with a single high-dose of melphalan (200 mg/m^2^) and 15 patients received a single 200 mg/m^2^ dose of melphalan combined with 1.3 mg/m^2^ velcade (6 and 3 days before and 1 and 4 days after).

#### Maintenance therapy

Nineteen patients received maintenance and consolidation therapy following ASCT. Of these, 13 were treated with 100–200 mg/day thalidomide, one received 25 mg/day lenalidomide, one had 3 million units/day interferon, three received treatment with 1.3 mg/m^2^ bortezomib (1, 4, 8 and 11 days following ACST) and one received progressive disease (PD) chemotherapy. However, the PD patient was unstable before ASCT. A further eight patients terminated their treatment due thalidomide intolerance, which manifested as neurotoxicity.

### Non-ASCT group

#### Induction chemotherapy

Ten patients received conventional chemotherapy and 18 patients were treated with bortezomib. The median number of chemotherapy treatments was two (range, 1–10). Following the induction therapy, there were nine cases of CR, 15 cases of nCR and four cases of VGPR.

#### Consolidation chemotherapy regimens

When all patients had received induction chemotherapy and achieved at least VGPR, they continued to receive consolidation chemotherapy, including 13 cases of conventional chemotherapy and 15 of bortezomib treatment. The median number of consolidation chemotherapy treatments was two (range, 1–8).

#### Maintenance chemotherapy

Following consolidation chemotherapy, 16 patients received 100–200 mg/day thalidomide as the maintenance therapy. Another 12 cases terminated their treatment, but once the disease recurred, they received chemotherapy again. There were no significant differences between the two groups of patients regarding treatment efficacy, and induction and maintenance therapy.

#### Follow-up and efficacy

All patients were followed up until October 30, 2011 to determine PFS and OS times. The PFS time was defined as the time interval between transplantation and disease progression or recurrence in the ASCT group, and the time from the first maximum effect following induction chemotherapy in the non-transplant group. The OS time was defined as the time interval from definitive diagnosis to mortality or termination during follow-up. The efficacy was defined as disclosed in the 2012 NCCN guidelines and was divided into CR, nCR, VGPR, PR, no change (NC), plateau, PD and CR recurrence (5). The total therapy (TT) was defined as bortezomib-based induction therapy prior to transplantation, two to four administrations of consolidation therapy once remission was achieved, autologous transplantation and administration of thalidomide as the maintenance therapy following the transplantation.

#### Statistical analysis

SPSS statistical software, version 16.0 (SPSS, Inc., Chicago, IL, USA) was used for statistical analysis. Measurement data was not normally distributed and presented as the median compared with the Mann-Whitney U test. Categorical data were compared with Fisher’s exact test and the survival curve was drawn by the Kaplan-Meier method. The log-rank test was used for univariate analysis. P<0.05 was considered to indicate a statistically significant difference.

## Results

### 

#### Impact of ASCT on treatment efficacy

All patients demonstrated recovered hematopoietic function and no transplant-related mortality occurred. Three months following transplantation, 19 patients achieved CR, which included nine cases of nCR and three of PR prior to transplantation. Additionally, five cases reached nCR, two cases reached PR and one had PD 3 months following transplantation. The efficacy in the 27 patients prior to transplantation was 92.6%, and the rates of CR, CR + nCR and PR were 25.9, 70.4 and 22.2%, respectively. The efficacy in the 27 patients 3 months after ACST, was 96.3%, and the rates of CR, CR + nCR and PR were 70.4, 88.9 and 7.4%, respectively. The CR incidence rate was significantly higher 3 months following ACST than that prior to transplantation (P<0.01; Table II).

#### Impact of ASCT on OS

**Figure 1.**
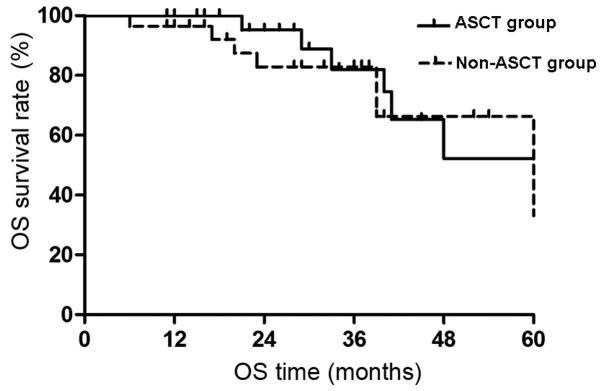
Comparison of OS between the different treatment groups. OS, overall survival; ASCT, autologous hematopoietic stem cell transplantation.

The median follow-up time for the 55 patients was 37 months (range, 11–99 months). In the ASCT group of 27 patients, there were five mortalities due to recurrence and a further mortality due to a severe lung infection 22 months following transplantation. In the non-ASCT group, there were six mortalities due to recurrence and one mortality from treatment-related causes out of the 28 patients. The ASCT group did not reach the median OS time at the final follow-up examination and the expected 5-year OS rate was 52.2±15.7%. The median OS time of the non-ASCT group was 60 months and the expected 5-year OS rate was 33.1±24.2%. The difference was not significant (P>0.05; Fig. 1).

#### Impact of ASCT on PFS

**Figure 2.**
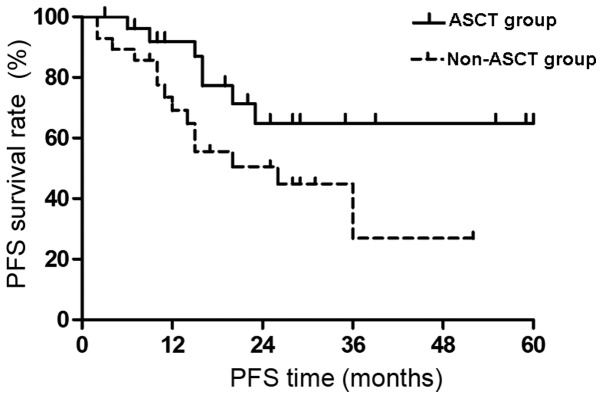
Comparison of PFS between the two treatment groups. PFS, progression free survival; ASCT, autologous hematopoietic stem cell transplantation.

The PFS survival curves of the two groups are shown in Fig. 2. There was no median PFS time in the ASCT group at the end of the follow-up period and the expected 4-year PFS was 64.9±11.1%. The median PFS time in the non-ASCT group was 20 months and the expected 4-year PFS was 26.9±11.7%. The PFS time was significantly extended in the ASCT group compared with that of the non-ASCT group (P<0.05).

#### Prognostic factors of the ASCT group

Univariate analysis of the prognostic factors for PFS and OS in the ASCT group are shown in Table III. The prognostic factors of PFS are as follows: i) PFS time was extended in patients receiving maintenance therapy following transplantation (P=0.01); ii) PFS time was significantly increased in patients with TT at the final follow-up examination (P=0.01); and iii) PFS time was increased in patients who were treated with bortezomib as the induction therapy, but without statistical significance. The prognostic factors of OS are as follows: i) OS time was extended in patients who received maintenance therapy following transplantation (P=0.008); ii) all patients undergoing TT survived; and iii) patients treated with bortezomib in the conditioning regimen showed an extended OS time, but without statistical significance. There were no clear relationships between the clinical indicators, age, gender, chromosome, disease type and staging with the PFS and OS times.

### Analysis of the prognostic factors in all patients

Univariate analysis of the prognostic factors of PFS and OS for all patients is shown in Table IV. Prognostic factors for PFS are as follows: i) PFS time was extended in patients <55 years old (P=0.008); ii) PFS time was extended in patients who underwent ASCT treatment (P=0.048); iii) PFS time was extended in patients receiving maintenance treatment, but without statistical significance; and iv) PFS time was increased in patients receiving TT (P=0.01). Furthermore, the prognostic factors for OS are as follows: i) OS time was extended in patients <55 years old (P=0.025); ii) OS time was extended in patients receiving ASCT treatment or TT, but without statistical significance; and iii) OS time was increased in patients receiving maintenance treatment (P=0.012).

## Discussion

MM accounts for 10% of malignant blood diseases and there has been significant progress in its treatment in recent years. High-dose chemotherapy with ASCT and novel targeted agents, such as thalidomide, bortezomib and lenalidomide, have been clinically applied, resulting in a significantly increased median survival time, particularly in young patients (6). However, thus far, MM remains incurable, the recurrence rate is high and it is difficult to obtain long-term survival. The present study examined the effects of ACST with novel targeted agents in patients with MM.

This study retrospectively analyzed the clinical data of 27 patients with ASCT from The First Affiliated Hospital of Soochow University. The majority of patients showed sensitivity to induction chemotherapy. The total effective rate was 92.6% and the CR rate was 25.9% prior to transplantation. Following transplantation, the efficacy was 96.3% and the CR rate increased to 70.4%. Therefore, ASCT further improved the response rate and remission quality, which is consistent with a previous study (7). The impact of ASCT on the prognosis of patients who achieved at least VGPR following induction chemotherapy, excluding the efficacy differences, showed that ASCT significantly extended the PFS time (P<0.05) and increased the median OS time (P>0.05). This may be due to the relatively short-term follow-up period (median, 37 months) and so the survival benefit may not have been presented. Barlogie *et al* were the first to demonstrate that ASCT with thalidomide increased the CR rate and PFS time, without increasing the OS time. When the median follow-up time reached 72 months, there was a correlation between CR rate and OS time, which was particularly significant in patients with cytogenetic abnormalities (8,9). Although there was no significant correlation between ASCT and OS time, the extension of PFS time is significantly important in improving patient quality of life (10).

A meta-analysis identified that in patients with MM treated by ASCT, the maximum efficacy of induction chemo-therapy was positively correlated with PFS and OS times (11). Harousseau *et al* compared the PD plan (bortezomib and dexamethasone) with that of VAD (vincristine, doxorubicin and dexamethasone) with regard to the efficacy and safety of induction therapy prior to transplantation. It was determined that before and after transplantation, the CR + nCR rate in the PD group was significantly increased compared with that of the VAD group (12). Various studies have also shown that induction therapy of novel agents, particularly a triple-drug combination, significantly improved the CR rate in patients with MM before and after transplantation (13–15). Furthermore, a phase II clinical study by Intergroupe Francophone du Myelome identified that the conditioning therapy of bortezomib in combination with high-dose melphalan, may further improve the CR rate following ASCT (16). In addition, studies have shown that for untreated or relapsed patients with MM, the maximum efficacy following treatment is the critical prognostic factor. A significant correlation has been identified between CR rate and PFS time, and the OS time has been observed to be significantly increased in patients with CR (17–19). In the present study, there was no improvement in the prognosis of patients who had received induction therapy and conditioning regimen with bortezomib, which may have been due to the short-term follow-up period. However, different results were observed in patients receiving TT. The TT plan is an integrated treatment that includes conditioning, transplantation, consolidation and maintenance therapies. TT1–TT3 data has demonstrated that as the intensity of the treatment increases, particularly in consolidation and maintenance therapies, the CR and OS rates significantly improve (20). Furthermore, in the present study, factors affecting the survival rate of patients with MM following hematopoietic stem cell transplantation were analyzed. No progress was identified in the PFS time in patients who received bortezomib; however, there was a positive correlation between PFS and OS times. This demonstrates the survival advantages of bortezomib and requires further study.

As there were no plateaus in the PFS and OS curves in patients with MM following transplantation, the residual myeloma cells will eventually result in relapse. Therefore, consolidation and maintenance therapies are recommended in order to prolong the release time. Thalidomide, used as maintenance therapy, aids in the inhibition of residual myeloma cells, thus improving the CR rate and extending the PFS and OS times (8,21,22). The present study analyzed the factors affecting survival and identified that the OS time in patients receiving maintenance therapy was significantly prolonged. In addition, PFS time was significantly increased in patients who had received transplant consolidation or maintenance therapy. Therefore, it is currently considered that combination chemotherapy using three types of novel agents, high-dose melphalan combined with intensive ASCT treatment, with similar induction therapy as consolidation chemotherapy and subsequent immunomodulatory agents as maintenance therapy, may be the optimal strategy for treating MM (23).

In conclusion, the present study indicated that ASCT improves the CR rate, PFS time, quality of life and potentially increases the OS rate following induction chemotherapy, in patients with MM. Novel therapeutic agents, such as bortezomib, further optimized ASCT treatment in induction and consolidation or maintenance therapies, subsequently benefiting patients with MM.

## Figures and Tables

**Table I.** Comparison of general clinical information from the two groups of patients with MM.

Groups	Cases	Age (years)	Gender (case)		Genotype (case)				ISS stage			DS stage		
			
			Male	Female	IgG	IgA	IgD	Light	I	II	III	I	II	III
Transplantation	27	51 (36–64)	18	9	13	12	1	1	2	13	12	2	2	23
Non-transplantation	28	54 (43–69)	15	13	17	5	1	5	1	12	15	2	5	21

**Table II.** Treatment efficacy comparison before and 3 months following transplantation (cases).

Transplantation groups	CR	nCR	PR	SD	PD	Relapse
Before	7	12	6	1	0	1
After	19^a^	5	2	0	1	0

a P<0.01 compared with before transplantation. CR, complete remission; nCR, non-complete remission; PR, partial remission; SD, stable disease; PD, progressive disease.

**Table III.** Univariate analysis of the prognostic factors for PFS and OS.

Prognostic factors	Cases	PFS	OS
Gender			
Male	18		
Female	9	0.566	0.320
Age (years)			
≥55	9		
<55	18	0.325	0.167
Genotype			
IgA+IgD	13		
Other	14	0.885	0.183
β2-MG (mg/l)			
≥5.5	13		
<5.5	14	0.105	0.211
Chromosome			
Abnormal	5		
Normal	22	0.801	0.652
Induction chemotherapy			
Traditional	12		
Bortezomib	15	0.626	0.524
Before transplantation			
Without CR	20		
With CR	7	0.476	0.460
Refractory or relapsed			
Yes	7		
No	20	0.174	0.138
Time span of transplantation and diagnosis (months)			
>6	11		
<6	16	0.563	0.743
Conditioning regimen			
HDM	11		
Bortezomib+HDM	16	0.471	0.219
CD34^+^ count (/kg)			
<3×10^6^	15		
≥3×10^6^	12	0.767	0.586
Efficacy after transplantation			
Without CR	8		
With CR	20	0.157	0.289
Maintenance treatment			
No	8		
Yes	19	0.010	0.008
Overall treatment			
No	13		
Yes	14	0.010	0.106

PFS, progression free survival; OS, overall survival; HDM: high-dose melphalan; CR, complete remission; β2-MG, β2-microglobulin.

**Table IV.** Univariate analysis of the prognostic factors for PFS and OS.

Prognostic factors	Cases	PFS	OS
Gender			
Male	33		
Female	22	0.293	0.613
Age (years)			
≥55	31		
<55	24	0.008	0.025
Genotype			
IgA+IgD	19		
Other	36	0.250	0.574
Bone destruction (sites)			
≥3	30		
<3	25	0.180	0.804
Hb (g/l)			
≤85	30		
>85	25	0.215	0.354
ALB (g/l)			
≤30	24		
>30	31	0.058	0.345
Cr (*μ*mol/l)			
≥177	12		
<177	43	0.366	0.702
Ca (mmol/l)			
≥2.17	30		
<2.17	25	0.534	0.675
LDH (U/l)			
≥200	28		
<200	27	0.434	0.435
β2-MG (mg/l)			
≥5	30		
<5	25	0.116	0.167
ISS stage			
III	27		
I+II	28	0.121	0.247
DS stage			
III	44		
I+III	11	0.437	0.294
Proportion of plasma cells (%)			
≥30	28		
<30	27	0.797	0.942
Chromosome			
Abnormal	8		
Normal	47	0.318	0.736
Induction chemotherapy			
Traditional	21		
Bortezomib	34	0.136	0.578
Efficacy of induction chemotherapy			
Without CR	37		
With CR	18	0.087	0.262
ASCT	27		
Efficacy of induction chemotherapy			
Non-ASCT	28	0.048	0.373
Maintenance treatment			
No	20		
Yes	35	0.143	0.012
Overall treatment			
No	41		
Yes	14	0.010	0.106

PFS, progression free survival; OS, overall survival; CR, complete remission; ASCT, autologous hematopoietic stem cell; Hb, hemoautologous hematopoietic stem cell; Hb, hemoglobin; ALB, albumin; Cr, creatine; Ca, blood calcium; LDH, lactate dehydrogenase; β2-MG, β2-microglobulin; ISS, International Staging System; DS, Durie-Salmon Staging System.
